# Effects of Naltrexone on Large-Scale Network Interactions in Methamphetamine Use Disorder

**DOI:** 10.3389/fpsyt.2019.00603

**Published:** 2019-09-03

**Authors:** Milky Kohno, Angelica M. Morales, Laura E. Dennis, Holly McCready, William F. Hoffman, P. Todd Korthuis

**Affiliations:** ^1^Department of Psychiatry, Oregon Health and Science University, Portland, OR, United States; ^2^Department of Behavioral Neuroscience, Oregon Health and Science University, Portland, OR, United States; ^3^Research and Development Service, Veterans Affairs Portland Health Care System, Portland, OR, United States; ^4^Methamphetamine Abuse Research Center, Oregon Health and Science University and Veterans Affairs Portland Health Care System, Portland, OR, United States; ^5^Mental Health Division, Veterans Affairs Portland Health Care System, Portland, OR, United States; ^6^Section of Addiction Medicine, Oregon Health and Science University, Portland, OR, United States

**Keywords:** naltrexone, resting-state functional magnetic resonance imaging, methamphetamine, striatum, functional connectivity

## Abstract

Naltrexone attenuates craving, and the subjective effects of methamphetamine and extended-release naltrexone (XR-NTX) reduces functional connectivity between regions of the striatum and limbic cortex. Naltrexone modulates neural activity at dopaminergic synapses; however, it is unclear whether naltrexone has an effect on large-scale brain networks. Functional networks interact to coordinate behavior, and as substance-use disorders are associated with an imbalance between reward and cognitive control networks, treatment approaches that target interactive brain systems underlying addiction may be a useful adjunct for behavioral therapies. The objective of this study was to examine the effect of XR-NTX on large-scale brain networks and to determine whether changes in network relationships attenuate drug use, craving, and addiction severity. Thirty-nine participants in or seeking treatment for methamphetamine-use disorder were enrolled in a clinical trial of XR-NTX between May 2013 and March 2015 (Clinicaltrials.gov NCT01822132). Functional magnetic resonance imaging (fMRI) and questionnaires were conducted before and after double-blinded randomization to a 4-week injection of XR-NTX or placebo. In the XR-NTX group, methamphetamine use was reduced along with a decrease in the coupling between executive control (ECN) and default mode (DMN) networks. As decoupling of ECN and DMN networks was associated with change in the severity of dependence, the results suggest that XR-NTX may modulate and enhance ECN attentional resources and suppress DMN self-referential and emotional processing. This study identifies the effect of naltrexone on changes in the intrinsic functional coupling of large-scale brain networks and provides a more systematic understanding of how large-scale networks interact to promote behavioral change in methamphetamine-use disorder.

## Introduction

Although methamphetamine (MA) is a highly addictive psychostimulant causing severe physical, neurological, and emotional disruptions (1), there are no FDA-approved medications for MA-use disorder (2). Psychosocial interventions, such as cognitive behavioral therapy, are the mainstay of treatment and are used to strengthen cognitive control over behaviors that promote drug use (2). The efficacy of behavioral interventions for MA use may be undermined by abnormalities in brain structure and function that are associated with impairments in executive functioning (3, 4) and linked to clinical features of addiction such as craving (5). Pharmacological interventions that alter neural network connectivity in individuals with MA-use disorders may have the potential to improve treatment outcomes.

Naltrexone, a competitive mu-opioid receptor antagonist, attenuates craving and subjective effects of MA in humans (6, 7). Although a 12-week study showed no differences between extended-release naltrexone (XR-NTX) and placebo on overall MA-use behavior (8), the effects of naltrexone on dopaminergic synapses to strengthen cognitive control could be a useful adjunct to behavioral therapy. Naltrexone, through downstream effects mediated by mu-opioid receptor antagonism, inhibits dopamine signaling in limbic regions. Consistent with naltrexone’s pharmacologic actions, we recently demonstrate that XR-NTX decreased connectivity between the nucleus accumbens, amygdala, hippocampus, and midbrain (9) using seed-based resting-state functional connectivity. Studies in healthy controls have demonstrated that changes in dopamine signaling affects the overall topography of resting-state networks that extend beyond dopamine terminal regions (10, 11). This study, therefore, examined the impact of XR-NTX on large-scale network interactions that may support behavioral approaches by strengthening cognitive control to abstain from MA use.

Advances in understanding the functional organization of brain systems suggest that a collection of interconnected brain areas work together to form functional networks that interact to coordinate behavior. Core networks include the Default Mode Network (DMN), which is comprised of the posterior cingulate cortex, temporal and medial prefrontal cortices and is associated with self-monitoring function and internal attention; the Executive Control Network (ECN), which includes the dorsolateral prefrontal cortex and posterior parietal cortices and is important for cognitive control; and the Salience Network (SN), which is comprised of the insula, anterior cingulate cortex, amygdala, ventral striatum, dorsomedial thalamus, hypothalamus, and substantia nigra/ventral tegmental area and is responsible for processing motivational stimuli and reward saliency (12). The goal of this study was to determine whether XR-NTX could alter the coupling between the ECN, DMN, and SN and to assess whether individual differences were associated with reductions in MA use and craving. We anticipated that XR-NTX would increase SN-DMN coupling, and this increase would be associated with reductions in MA craving. Prior work has also demonstrated that individuals with MA use disorder have greater coupling between the ECN and DMN than control participants (13). Effective treatments for MA-use disorders may ameliorate abnormalities in network correlations; therefore, we hypothesize that XR-NTX would decrease coupling between the ECN and DMN.

## Materials and Methods

### Participants

Thirty-nine participants were enrolled in a randomized, double-blind, placebo-controlled clinical trial of XR-NTX (Vivitrol, Alkermes). Participants were recruited from community-based treatment programs and primary care clinics in Portland, Oregon, USA, between May 2013 and March 2015 and were included in a previous study examining seed-based resting-state connectivity (9). Inclusion criteria were a DSM-IV diagnosis for methamphetamine dependence, no other substance dependence except tobacco and/or nicotine dependence, no history of psychiatric disorder except depression and/or post-traumatic stress disorder, aspartate transaminase (AST) and alanine transaminase (ALT) < 5 times the upper limit of normal, between the ages of 18 and 55 years, right-handed, English speaking, and free of drugs and alcohol >72 h and no more than 6 months prior to study assessments. Exclusion criteria included opioid use in the last 30 days or opioid dependence in the past 5 years, asensitivity to naltrexone, PLG (polylactide-co-glycolide), carboxymethylcellulose, or any other diluent components, a potential need for opioid analgesics during study period, pregnancy, magnetic resonance imaging (MRI) contraindications, or serious medical illness in the past 30 days.

### Study Design

The study was approved by the Oregon Health and Science University and Veterans Affairs Portland Health Care System Joint Institutional Review Board, and all participants provided written informed consent. At baseline, participants underwent resting-state functional MRI and completed survey assessments (Visit 1). Using a double-blind design, participants were randomized to XR-NTX (n = 19) or placebo (n = 20) groups based on the output from a computerized random number generator. To reduce issues with study drug adherence challenges associated with daily-dosed oral naltrexone, both the XR-NTX and placebo conditions involved a single 4-week injection, which was donated by the manufacturer. Survey and brain imaging assessments were repeated 4 weeks after baseline scans (Visit 2).

#### Neuropsychiatric Assessment

A Mini International Neuropsychiatric Interview (MINI) (14) was conducted to confirm substance dependence diagnoses and psychiatric disorders and the Addiction Severity Index-lite (ASI-lite) (15, 16) was used to assess past 30-day substance use. MA craving was measured with a visual analog scale (VAS) ranging from 0 (no craving) to 100 (most intense craving possible) (17). The Substance Dependence Severity Scale (SDSS), which is sensitive to change in clinical status, was administered to assess the severity of substance use (18).

#### MRI Imaging Acquisition

Imaging was performed on a 3 Tesla Siemens TIM Trio MRI scanner. A localizer scan was acquired in order to guide slice alignment during anatomical and functional scans. A T_2_*-weighted image was acquired using an echo planar imaging scheme (EPI; 24 slices, 4-mm thick, gap width = 1 mm, TR/TE/α = 2,000 ms/38 ms/80°, matrix = 128 × 128, FOV = 240 × 240 mm, 170 volumes, in-plane pixel size of 1.9 mm^2^), while subjects stared at a white cross on a black screen for 6 min. One high-resolution T_1_-weighted magnetically prepared rapid acquisition gradient echo (MPRAGE; 76 slices, 1-mm thick, TR/TE/TI/α = 2,300 ms/3.4 ms/1,200 ms/12°, FOV = 224 × 256 mm) was acquired for co-registration with functional images and statistical overlay.

#### Resting-State Processing and Group-Level Analyses

**Figure 1:**
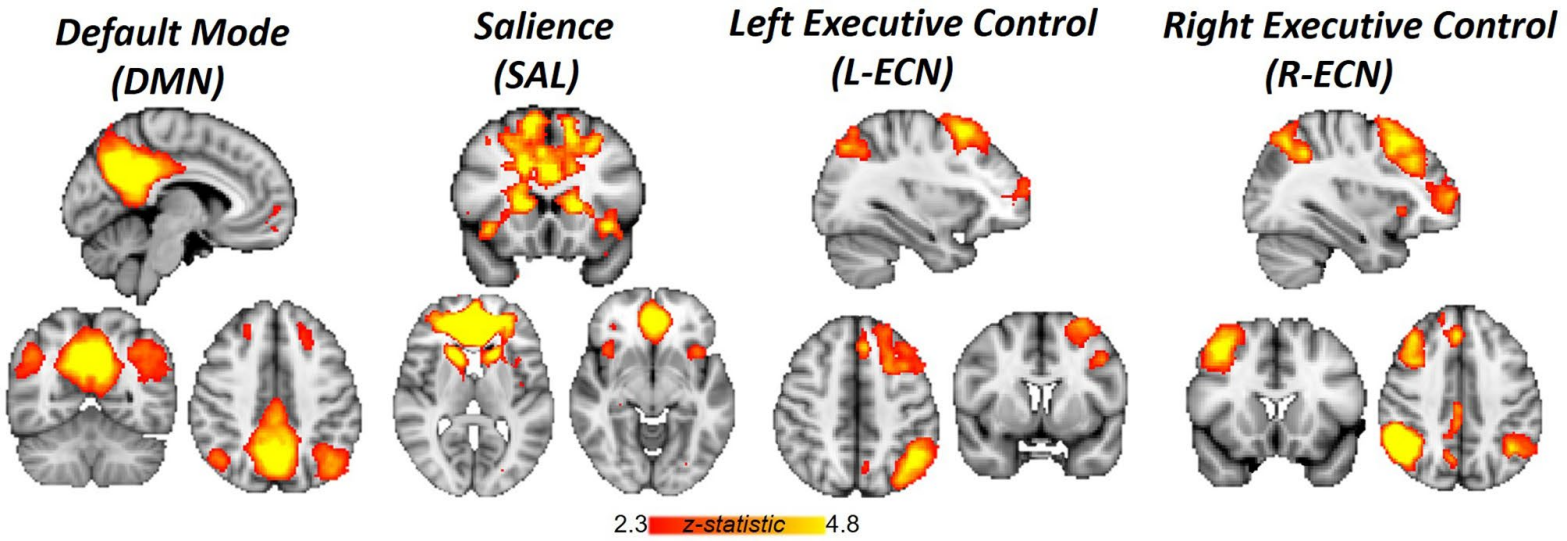
Networks identified by independent component analysis. Spatial maps generated with group ICA and cross-correlated to resting-state template masks include Default Mode Network, Salience Network, and Left and Right Executive Control Network.

Image analysis was performed using FSL 5.0.2.1 (www.fmrib.ox.ac.uk/fsl). Images were skull-stripped, spatially smoothed [5-mm full width at half maximum (FWHM) Gaussian kernel], and realigned to compensate for motion (19). Automatic Removal of Motion Artifacts (AROMA) was then used to reduce motion-induced signal variation using independent component analysis (ICA) with a classifier that uses two temporal and two spatial features to remove motion artifacts. To identify large-scale resting-state networks across all subjects, cleaned outputs from AROMA underwent ICA analysis with Multivariate Exploratory Linear Optimized Decomposition into Independent Components (MELODIC). The number of components generated was not restricted, and 67 group-average independent components were identified. The spatial maps of these independent components were cross correlated with resting-state templates (20) to identify ECN, DMN, and SN networks (**Figure 1**). FSL’s dual regression was used to regress the group spatial maps of the ECN (left and right), DMN, and SN networks for each subject to identify subject-specific spatial maps of each network. For each subject and scan, average time courses were extracted for ECN, DMN, and SN networks. Time courses from the ECN, DMN, and SN for each scan were imported into R (version 3.3.2) and used to generate between pairwise Pearson correlations between each network for each subject. Correlation coefficients were converted to z-scores *via* Fisher’s transformation.

#### Statistical Analysis

Student’s t-tests and Fisher’s exact tests, where appropriate, were used to compare groups in baseline demographic and clinical variables (**Table 1**). Repeated measures ANOVAs were used to examine the effects of XR-NTX on craving and MA use. The main effect of treatment (XR-NTX or Placebo) and time (Visit 1 or Visit 2) and the interaction of treatment and time were examined on each measure separately. To assess changes in large-scale resting-state network correlations, two-way repeated measures ANOVAs on pairwise correlations between the ECN, DMN, and SN were tested for main effects of group (XR-NTX and Placebo), time (Scan 1 and Scan 2), and the interaction of group by time in SPSS 22. Bivariate correlations were conducted to explore the association between changes in network coupling associated with XR-NTX administration and changes in craving, frequency of MA use, and substance dependence severity between Visit 1 and Visit2.

**Table 1:** Participant Characteristics.

	Placebo (n = 20)	NTX (n = 19)	p-value
Age (years) ^a^	36.47 ± 10.06	38.68 ± 9.30	0.49
Sex (M/F) ^b^	15/5	15/4	0.77
Education	12.63 ± 0.83	12.78 ± 2.13	0.78
Craving			
Baseline	23.84 ± 27.27	32.83 ± 27.69	0.33
Follow-up	18.63 ± 25.16	20.06 ± 25.63	0.87
MA use: Days in the last 30 Baseline Follow-up			
	3.55 ± 6.42	5.05 ± 6.93	0.51
	3.87 ± 7.09	1.56 ± 3.45	0.23
Substance Use Severity Scale			
Baseline	5.37 ± 3.76	8.17 ± 4.30	0.04
Follow-up	3.84 ± 4.25	3.42 ± 4.23	0.76
Smoking Number of smokers ^b^	17	14	0.34
Positive HIV Status ^b^	5	6	0.65

^a^Data shown are means ± Standard Deviations.

^b^Data analyzed with Chi-squared test (X^2^).

### Results

#### Participant Characteristics

A total of 220 individuals were screened for the study, and 104 were eligible for participation. The most common reasons for exclusion at pre-screening were polysubstance use, abstinence from MA for over 6 months, and MRI contraindications. Of the 104 eligible participants, 52 were randomized (50% of those who were eligible; 23.6% of those screened). Three eligible participants declined randomization. Of those randomized, 39 completed baseline and follow-up assessments that were available for analysis. Reasons for exclusion from analysis of those randomized included scheduling conflicts/no-shows and MRI confounds.

At baseline, groups were well-matched on demographic variables (**Table 1**). Participants did not differ by mean age (Placebo: 36.47 years; XR-NTX: 38.68 years, p = 0.49), sex (Placebo: 75% men; XR-NTX: 79% men, p = 0.772), mean years of education (Placebo: 12.63 years; XR-NTX: 12.78 years, p = 0.78), or smoking status (Placebo: 85%: XR-NTX: 74%, p = 0.34). There were no significant group differences in MA use in the 30 days prior to study enrollment (Placebo: 3.55 days; XR-NTX: 5.05 days, p = 0.444), craving for MA indexed by the VAS (Placebo: 23.84; XR-NTX: 32.83, p = 0.327), or HIV status (XR-NTX: 25%; Placebo group: 32%, p = 0.65) but significant differences in SDSS (Placebo: 5.37; XR-NTX: 8.17, p = 0.04). One HIV-positive subject in each group had no current or past history of taking stable antiretroviral therapy, but all other HIV-positive patients were taking stable antiretroviral therapy prior to and during the study.

#### Change in Methamphetamine Use, Substance Dependence Severity, and Craving

On average, the number of days in the past 30 days of self-reported MA use decreased from 5.05 to 1.56 in the XR-NTX group but increased in the Placebo group from 3.55 to 3.87 in the Placebo group. The repeated measures ANOVA resulted in a significant time by treatment interaction (p = 0.03), with the XR-NTX group showing greater reductions in MA use compared to the Placebo group. Mean craving scores decreased from 32.83 to 20.07 in the XR-NTX group and from 23.84 to 18.63 in the Placebo group. There were no significant time by treatment interactions on craving and (p = 0.52) or on SDSS (p = 0.13).

#### Changes in Coupling Between ECN, DMN, and SN

**Figure 2:**
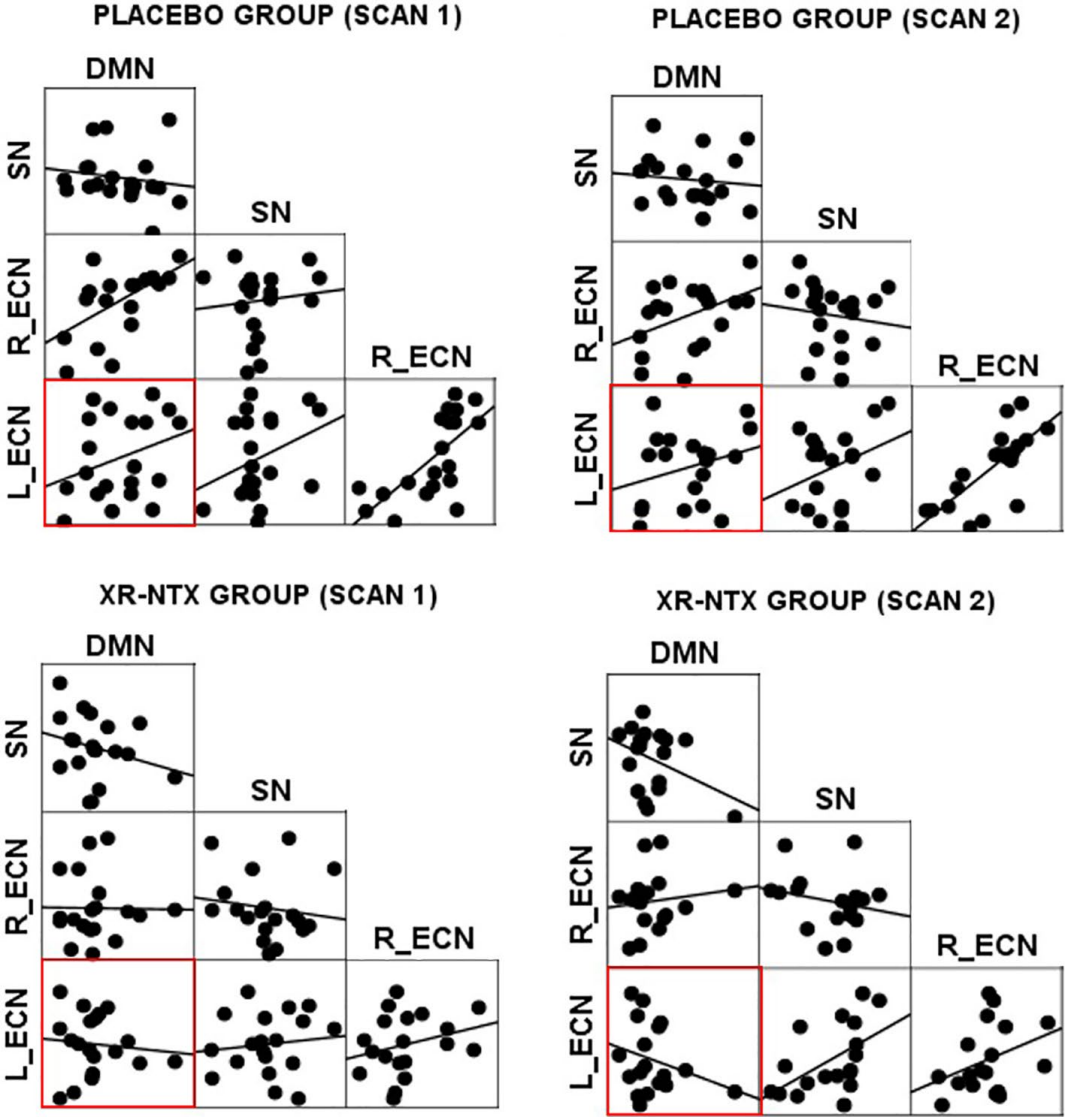
Network correlations. Scatter plots depict the relationships between networks in each group for each scan.

**Figure 3:**
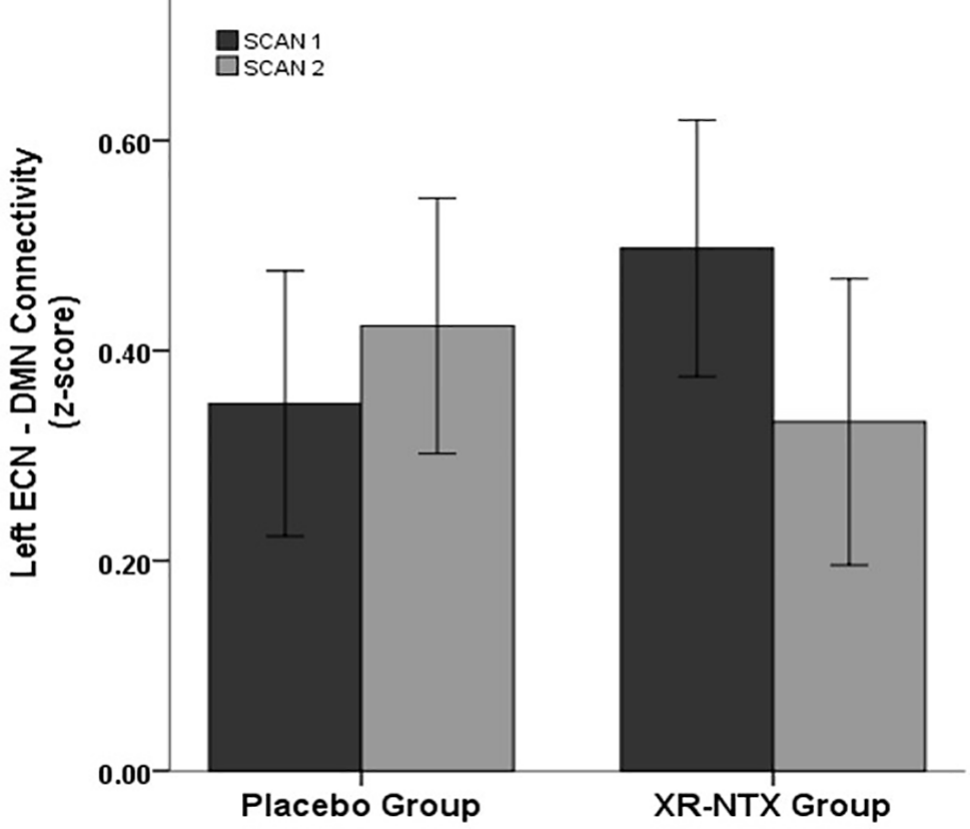
Change in network correlations. Left ECN–DMN correlation. The XR-NTX group show significant reductions between Scan 1 and Scan 2 in Left ECN–DMN coupling compared to the placebo group (p = 0.002).

Correlations between networks for each subject and scan are depicted in **Figure 2** for illustrative purposes. In the repeated measures group analyses, a significant interaction of group and time was seen in the correlation between Left ECN and DMN connectivity (p = 0.002, corrected for multiple comparisons) (**Figure 3**), with no significant differences in correlation at Time 1 (p > 0.05) or at Time 2 (p > 0.05). The group by time interaction remained significant (p = 0.01) after controlling for the group difference in SDSS. There were no significant group by time interactions on the connectivity between SN and Left ECN (p = 0.086), SN and Right ECN (p = 0.280), DMN and SN (p = 0.704), DMN and Right ECN (p = 0.898), or Left ECN–Right ECN (p = 0.424).

#### Relationship between resting-state functional connectivity and clinical outcome measures

**Figure 4:**
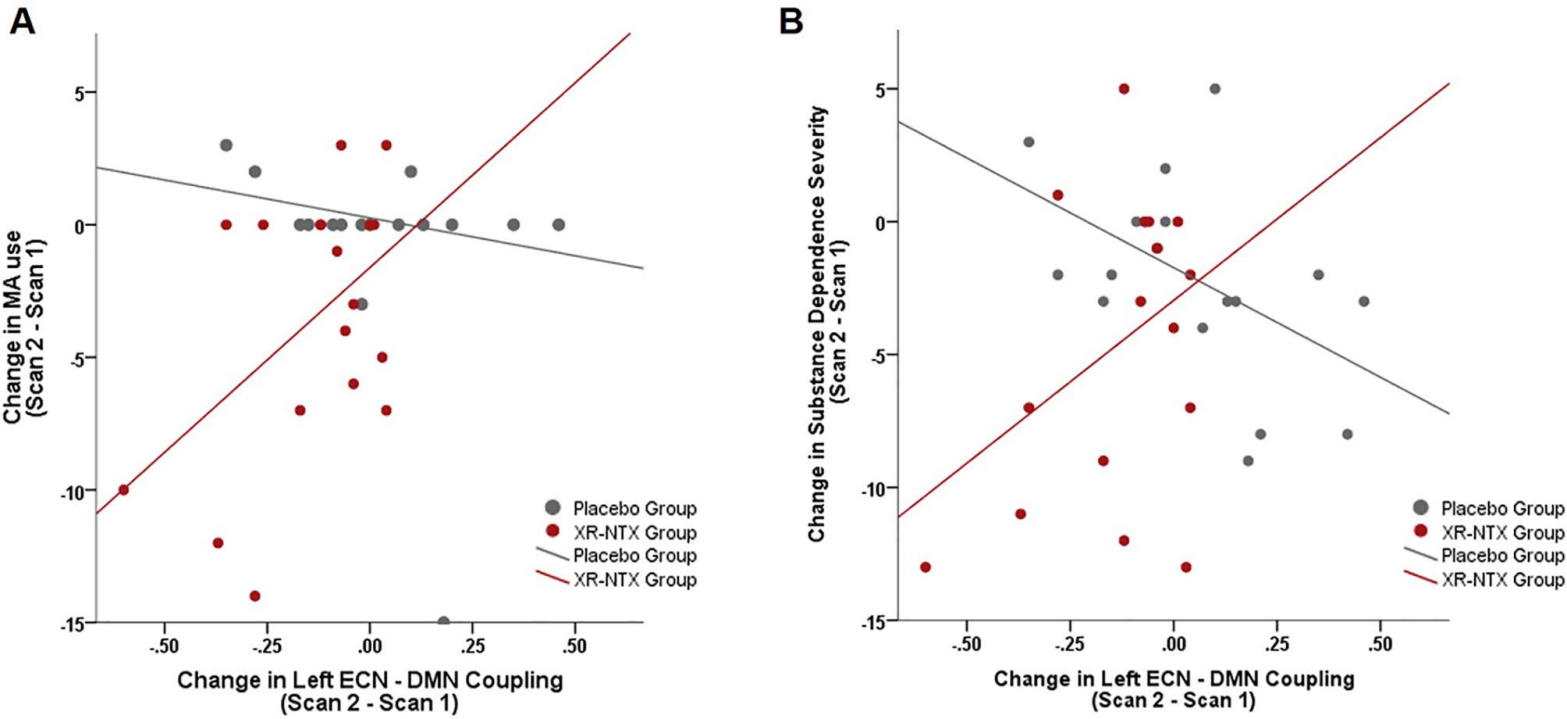
Change in Left ECN–DMN correlations is associated with change in MA use and Substance Dependence Severity. **(A)** In the XR-NTX group, individuals with greater reductions in network correlations exhibit greater reductions in MA use between Scan 1 and Scan 2 with opposite effects in the Placebo Group (p = 0.019). **(B)** In the XR-NTX group, individuals with greater reductions in network correlations exhibit greater reductions in Substance Dependence Severity scores between Scan 1 and Scan 2 with opposite effects in the Placebo Group (p = 0.014).

As a group by time interaction on Left ECN and DMN correlations was detected, we explored how changes in Left ECN–DMN coupling affect MA use, craving, and substance dependence severity. When examining the relationship between change in MA use and change in network coupling, we found a significant group interaction (**Figure 4**, p = 0.04); where the XR-NTX group showed a positive relationship between change in Left ECN–DMN network coupling and change in MA use, while the Placebo group showed a negative relationship. Similarly, the groups differed in the relationship between Left ECN–DMN network change and substance dependence severity (**Figure 4**, group by connectivity interaction: p = 0.014), where the XR-NTX group showed a positive relationship and the Placebo group showed a negative relationship. Results remained significant after controlling for duration of MA abstinence (p = 0.003), confirming that changes in ECN–DMN coupling was an effect of group and not reductions in MA use. There were no significant interactive effects of group and connectivity on craving (p > 0.05).

## Discussion

This study provides novel evidence indicating that XR-NTX modulates connectivity between large-scale brain networks in individuals with MA dependence. Specifically, XR-NTX reduced coupling between the Left ECN and DMN, which was related to a reduction in MA use and severity of substance dependence. These results are consistent with the role of the ECN in directing attention toward relevant stimuli, flexibly responding to shifting conditions and executing goal-directed behavior (12) and the DMN in processing internal states and episodic memory (21). Given that the activity of ECN and DMN are often anti-correlated and the decoupling from the DMN enables the ECN to allocate attentional resources and to flexibly switch attention in the face of changing cognitive demand, XR-NTX-induced decreases in network coupling may enhance network dynamics to strengthen cognitive resources to limit drug use. In a recent study, baseline DMN connectivity was a predictive factor for treatment outcome in obsessive–compulsive disorder, suggesting that brain connectivity patterns may reflect plasticity of networks that facilitate cognitive and behavioral change (22). As cognitive behavioral therapy requires cognitive flexibility to regulate craving and withdrawal, XR-NTX may be a useful adjunct to treatment to induce network changes that enable plasticity of executive control networks to function without constraint of self-referential DMN activity during abstinence.

Our findings provide support that medication-induced alterations in dopamine signaling impact resting-state connectivity between the ECN and DMN in individuals with a MA dependence. In particular, one study found that MA-dependent individuals with and without MA-induced psychosis had greater ECN–DMN connectivity than control participants (13). In the group of individuals with MA-induced psychosis, lower ECN-DMN connectivity was associated with longer exposure to antipsychotic medications such as the dopamine D2 receptor antagonist, haloperidol. In addition, this study showed that the duration of antipsychotic medication and ECN–DMN connectivity remained significant when controlling for duration of abstinence from MA, suggesting that changes in ECN–DMN connectivity are related to the medication as opposed to reductions in MA use, which is consistent with our findings. Since both antipsychotic medication and naltrexone impact multiple neurotransmitter systems, studies combining resting-state functional connectivity with positron emission tomography to assess neurotransmitter release or receptor density will be useful for uncovering the molecular underpinning of ECN–DMN interactions.

The association between XR-NTX-induced changes in ECN–DMN coupling, MA use, and substance dependence severity suggest that interventions that successfully alter ECN–DMN connectivity may be especially useful for treating MA-use disorders. In our sample, individuals with smaller changes in ECN–DMN correlations after XR-NTX used MA more frequently, suggesting that for these individuals, identifying other medications that have a greater impact on ECN–DMN coupling may improve treatment outcomes. For example, modafinil has been shown to increase negative coupling between the ECN and DMN and improve cognitive performance in individuals with alcohol-use disorder (23). Although some studies suggest that modafinil is not effective for decreasing MA use (24), these findings may be attributable to heterogeneous medication responses at the individual level.

Studies have shown a positive effect of naltrexone in reducing MA craving (6, 7); however, a recent study showed no differences in MA use after 12 weeks of placebo or XR-NTX (8). Mixed results could be attributed to the positive HIV status in the majority of subjects in the latter study, or it is possible that the effects of naltrexone on MA abstinence are time dependent. In the study where MA use did not differ after 12 weeks between the placebo and XR-NTX groups, the XR-NTX group did show a substantial increase in the proportion of participants abstaining from MA in weeks 3, 4, and 5 with no change in the placebo group. As our results show dynamic change in network interactions during this short window, it is possible that this network change may facilitate early MA abstinence. It is unclear whether groups in our study would have converged in MA use or network dynamics after 12 weeks of treatment, but perhaps early network changes coupled with other treatment approaches or cognitive behavioral therapy can help strengthen cognitive control to limit MA use.

Our findings should be interpreted with consideration of the following potential limitations. Although the difference between placebo and XR-NTX on changes in ECN–DMN coupling remained significant after controlling for frequency of MA use, more research is needed to determine whether changes in ECN–DMN coupling precede and causally impact MA use. Furthermore, our sample was relatively small, precluding our ability to examine whether there were sex by treatment interactions on large-scale network dynamics or clinical variables. In addition, future studies could take a data-driven approach to identify spatially constrained regions that drive alterations in large-scale network interactions. Last, although the placebo and XR-NTX groups were matched for cigarette use, smoking has been linked to abnormalities in large-scale network dynamics (25). More research is needed to determine how XR-NTX impacts ECN–DMN coupling in individuals with MA-use disorders who do not smoke cigarettes; however, since the vast majority of individuals with MA-use disorder also smoke cigarettes (26), the design of our study may have greater generalizability.

## Conclusion

This study provides new evidence of the effect of naltrexone on large-scale brain network dynamics. As the independence of the ECN from other network activity is thought to enable flexible resource allocation during high cognitive demand, the XR-NTX-induced reduction in network coupling between ECN and DMN may facilitate decreases in MA use. Network modifications can facilitate cognitive and behavioral control; however, substance-use disorders are accompanied by a number of psychosocial factors that need to be addressed to maintain recovery. Although XR-NTX-induced changes in network dynamics can support behavioral changes, it is likely that a combination of approaches that target neural function and cognitive behavioral changes may provide the most therapeutic benefit. This study provides new information on how network changes can affect MA dependence and use. Future studies are required to understand whether XR-NTX-induced brain changes coupled with behavioral therapy would enhance recovery. Conducting clinical trials with cross-over designs to examine the extent to which various medications can impact relevant biomarkers such as ECN–DMN coupling that facilitate behavioral therapy may be useful for tailored treatments that consider an individual’s unique pharmacological response.

## Data Availability

The datasets generated for this study are available on request to the corresponding author.

## Ethics Statement

The studies involving human participants were reviewed and approved by OHSU IRB. The patients/participants provided their written informed consent to participate in this study.

## Author Contributions

PTK and WH designed and implemented the study. LD and HM managed and oversaw the study implementation. MK conducted the analysis and drafted the manuscript. MK and AM contributed to data interpretation. All authors took part in the revision of the manuscript and approved the article.

## Funding

The study was funded by the U.S. National Institutes of Health, National Institute on Drug Abuse (R21DA033182, P50DA018165 07, UG1DA015815, T32DA007262, and T32AA007468), National Center for Research Resources (NCRR), a component of the National Institutes of Health (NIH) and NIH Roadmap for Medical Research [1 UL1 RR024140 01 Oregon Clinical and Translational Research Institute (OCTRI)], and Department of Veterans Affairs Clinical Sciences Research and Development Merit Review Program, I0CX001558 (WH) and Development Career Development Award IK2CX001790 (MK), Oregon Health and Science University Collins Medical Trust Award APSYC0249, and Medical Research Foundation of Oregon APSYC0250. The manufacturer, Alkermes, donated extended-release naltrexone and placebo injections for use in this trial.

## Disclaimer

The contents of this paper do not represent the views of the U.S. Department of Veterans Affairs or the United States Government.

## Conflict of Interest Statement

The authors declare that the research was conducted in the absence of any commercial or financial relationships that could be construed as a potential conflict of interest.
